# Barriers and facilitators for the implementation of the CombiConsultation by general practitioners, pharmacists and practice nurses: a qualitative interview study

**DOI:** 10.1007/s11096-023-01597-7

**Published:** 2023-05-30

**Authors:** Valérie A. M. Meijvis, Mette Heringa, Henk-Frans Kwint, Niek J. de Wit, Marcel L. Bouvy

**Affiliations:** 1 SIR Institute for Pharmacy Practice and Policy, 2331 JE Leiden, The Netherlands; 2 Department of Pharmaceutical Sciences, Utrecht University, Utrecht, The Netherlands; 3 Department of General Practice, Julius Centre for Health Sciences and Primary Care, University Medical Centre Utrecht, Utrecht, The Netherlands

**Keywords:** CombiConsultation, Community pharmacist, General practice, Pharmaceutical care, Primary care

## Abstract

**Background:**

The CombiConsultation is a consultation with the pharmacist for patients with a chronic condition, aligned with the periodic consultation with the practice nurse or general practitioner. Implementation requires adjustments in the working methods of these healthcare providers and therefore behavioural changes.

**Aim:**

The aim of this study was to identify the barriers and facilitators that determine the behavioural changes by pharmacists, general practitioners and practice nurses required for the implementation of the CombiConsultation.

**Method:**

Ten community pharmacists, 5 practice nurses and 5 general practitioners were sampled from practices enrolled in the CombiConsultation study. Their views regarding the implementation of this clinical pharmacy service were explored using interviews based on the 14 domains of the Theoretical Domains Framework (TDF), which are linked to the Capability-Opportunity-Motivation-Behaviour-model. Barriers and facilitators in the domains were assessed by content analysis.

**Results:**

Twelve barriers and 23 facilitators were found within 13 TDF domains with high agreement between the healthcare providers. Important facilitators for implementation were the pharmacists’ expertise in pharmacotherapy (capability), access to medical data and physical proximity between professional practices (opportunity). Barriers were pharmacists’ insufficient consultation- and clinical-reasoning skills (capability), insufficient staff (opportunity) and reimbursement and lack of coordination among all involved healthcare providers (motivation).

**Conclusion:**

All healthcare providers are motivated to implement the CombiConsultation. An existing collaborative practice, with a clear and accepted professional role of the pharmacist is essential. Training of pharmacists in consultation and clinical-reasoning skills can be beneficial, as well as arrangements on the consultation logistics, and reimbursement.

**Supplementary Information:**

The online version contains supplementary material available at 10.1007/s11096-023-01597-7.

## Impact statements

- The CombiConsultation can contribute to the acceptance of the pharmacist's professional role.
- To perform the CombiConsultation optimally, pharmacists should improve their consultation and clinical-reasoning skills.
- The proximity of professional practices is conducive to interprofessional collaboration.

## Introduction

Worldwide, approximately one in three of all adults suffer from multiple chronic conditions. Therefore, the prevention and management of these noncommunicable diseases has been made a global priority [1]. In the Netherlands, half of the population has at least one chronic disease and 3 out of 10 people have multiple chronic conditions. Due to aging of the population, these numbers are expected to increase in the coming years [2] and the issue of staffing shortages in healthcare will become one of the biggest challenges [3]. Patients with multiple chronic conditions often use multiple medications (polypharmacy). In patients with polypharmacy (defined as ≥ 5 medicines in long-term use [4]) the risk of adverse drug reactions, suboptimal use and effects of medication are substantially increased, resulting in increased risk of health care utilization [5, 6] and higher total healthcare expenditures [7] For this reason, adequate medication management has become increasingly important [5, 8]. Several programmes have been developed to improve pharmacotherapy in older adult patients [9–11], and clinical medication review has been successfully implemented for older adult patients with polypharmacy in the Netherlands. However, for other patient groups, such as those who are younger and not (yet) polymedicated, no specific pharmaceutical services are presently offered.

We therefore have developed an alternative service for patients aged 18 or over, with a chronic condition and at least one medicine in use: the CombiConsultation. It involves a consultation with the patient lasting 15–20 min, aligned with the check-up with the practice nurse (PN) or the general practitioner (GP). During this consultation, the community pharmacist (CP) focusses primarily on setting personal health-related goals together with the patient and identifies drug-related problem; goals and interventions are evaluated after a few weeks (often 2–4 weeks, depending on the goal set) [12]. By consulting the patient about his complaints, a joint health-related goal can be set. This allows the CP to contribute in chronic care programs to provide patient oriented care regarding medication and thus supplements the care provided by the PN and GP.

Implementation of the CombiConsultation requires adjustments in the working methods of the CP, PN and GP, therefore involving behavioural change. Changing professional behaviour is complex and requires an understanding of the key factors that influence it, including capability, opportunity and motivation [13].

### Aim

The aim of this study was to identify the barriers and facilitators that can influence the behavioural change of CPs, GPs and PNs in the implementation of the CombiConsultation.

### Ethics approval

This study was exempted from formal medical ethical approval by the Medical Ethical Committee of the University Medical Centre Utrecht (METC protocol number 17–873/C) and the research protocol was approved by the Institutional Review Board of UPPER, Division of Pharmacoepidemiology and Clinical Pharmacology, Utrecht University (UPF1706; January 2018). All participants provided informed consent for the use of the data collected for the purpose of this study. Videos and audio fragments were coded and stored on a secure server. We followed the reporting recommendations of the consolidated criteria for qualitative research (COREQ) [14].

## Method

### Setting

We performed a qualitative interview study within a prospective intervention study ‘the CombiConsultation’, which was performed between January 2017 and July 2019 in 21 community pharmacies and associated GP practices in the Netherlands.

#### The CombiConsultation study

The intervention consisted of a CombiConsultation performed by a CP in collaboration with a PN or GP. The CP focussed on potential health-related complaints related to the chronic condition for which the patient had an appointment with the PN or GP. All CPs had, with the patient’s consent, access to medical data (at least conditions and laboratory values). The CP set personal health-related goals together with the patient and identified drug related problems (DRPs). After the consultation, the CPs discussed the DRPs with the PN or GP and recommendations could be implemented. A few weeks later, the CP or PN/GP evaluated the implementation of suggested recommendations and whether the personal health-related goals had been attained [12]. During the study, 834 CombiConsultations were performed. The median number of consultations per pharmacy was 29 (range 2–106) [15].

### Study design

This qualitative study comprised semi-structured interviews with 10 CPs and 10 healthcare providers from the general practice (5 GPs and 5 PNs) who participated in the prospective evaluation of the CombiConsultation. The interviews aimed to explore their personal views regarding the barriers and facilitators that could affect the implementation of the CombiConsultation.

### Data collection and participants

Interview guides tailored for GPs, PNs and CPs were developed by 2 authors (VM and MH) who are pharmacists/researchers and had training in qualitative research. The interview guides were based on the Theoretical Domains Framework (TDF) (Supplementary information 1). The TDF contains 14 domains that allow a comprehensive theoretical assessment of implementation problems. To investigate behavioural change, these domains were linked to the components of the Capability–Opportunity–Motivation–Behaviour (COM-B) model [16]. The guides were discussed with the research team until a final version was compiled, consisting of 20 (CP), 21 (PN) and 20 (GP) main questions in all domains of the TDF. The initial interview guide was tested with initial pilot interview with a CP, GP and PN who participated in the intervention study the ‘CombiConsultation’. No major changes were necessary; therefore, these interviews were also included in the analysis. Data saturation was defined as the point at which no new main codes emerged and was checked after the tenth (CP) and fifth (GP and PN) interview. [17].

Data collection was performed between July and September of 2019. Ten CPs, 5 PNs and 5 GPs were recruited using purposive sampling based on their location and number of consultations performed (Table 1). All invited healthcare providers were willing to participate. Due to participation in the intervention study, most of them knew the researchers (VM and MH) and the purpose of their study. Participants received €50 for participation. Interviews were performed by VM (trained in conducting interviews) and/or Master student pharmacy (WN, conducted interviews after training and observation). Interviews were in Dutch and face to face (in the pharmacy, general practice or research institute) or by telephone, ensuring sufficient privacy.

**Table 1:** Characteristics of pharmacists, general practitioners and practice nurses

	Gender	Years of experience	GP practice and pharmacy in the same building?	Clinical setting of CombiConsultation (consultation with pharmacist)	Area	Mode of interview	Number of CombiConsultations performed
*Pharmacists*							
1	Female	14 years	Co-located	Pharmacy	Rural	Face to face	10
2	Female	10 years	Co-located	GP practice	Urban	Face to face	76
3	Female	16 years	Co-located	GP practice	Urban	Face to face	81
4	Female	7 years	Co-located	Pharmacy and GP practice	Urban	Telephone	44
5	Female	20 years	Co-located	Pharmacy and GP practice	Urban	Face to face	11
6	Male	25 years	Co-located	Pharmacy	Rural	Telephone	37
7	Male	21 years	Co-located	GP practice	Urban?	Telephone	98
8	Female	9 years	Co-located	Pharmacy	Rural	Telephone	2
9	Male	2 years	Co-located	Pharmacy	Urban	Face to face	32
10	Female	13 years	Separate	GP practice	Urban	Face to face	67
*General practitioners*							
1	Female	27 years	Co-located	Pharmacy	Rural	Telephone	37
2	Female	12 years	Co-located	GP practice	Rural	Telephone	29
3	Male	25 years	Separate	GP practice	Urban	Face to face	60
4	Female	11 years	Co-located	Pharmacy	Urban	Face to face	32
5	Female	12 years	Co-located	Pharmacy	Rural	Face to face	10
*Practice nurses*							
1	Female	20 years	Co-located	GP practice	Urban	Telephone	15
2	Female	12 years	Co-located	Pharmacy and GP practice	Urban	Telephone	20
3	Female	4 years	Separate	Pharmacy and GP practice	Urban	Telephone	18
4	Female	17 years	Co-located	Pharmacy	Urban	Telephone	61
5	Female	13 years	Co-located	GP practice	Rural	Telephone	22

Collaborating couples: CP1 and GP5, CP6 and GP6, CP9 and GP4

### Data analysis

All interviews were audio-recorded and transcribed verbatim. NVivo qualitative data analysis software (version 12 Pro, QSR International) was used for data analysis. Interview transcripts were analyzed using content analysis [18], and the barriers and facilitators perceived by CPs, GPs and PNs as being relevant for the implementation of the CombiConsultation were categorized within the TDF domains.

Initially separate analyses were performed for CPs, GPs and PNs. Transcripts were read repeatedly to ensure familiarization with the data. Thereafter, initial codes were assigned and linked to the TDF by VM and WN independently. Differences and uncertainties were resolved by consensus through discussions involving a third researcher (MH) with experience in using the TDF. This process resulted in a final coding scheme for the 3 groups of healthcare providers. The resulting barriers and facilitators were discussed with the research team to ensure consensus. Finally, the barriers and facilitators of the different healthcare providers were compared, and overlapping factors were combined when possible. Barriers and facilitators were structured per TDF domain according to the COM-B model.

## Results

Including the pilot interview, a total of 20 interviews were conducted (10 CPs, 5 PNs and 5 GPs). Data saturation was reached after the 10th (CP) and 5th (GP and PN) interview. The 20 participants were primarily female (*n* = 16, 80%) and possessed a mean clinical experience of 14.5 years (Table 1). The duration of the interviews ranged from 23 to 67 min. The median durations of interviews for CPs, GPs and PNs were 30, 30 and 23 min, respectively.

Using content analysis, the barriers and facilitators to the implementation of the CombiConsultation perceived by CPs, GPs and PNs were categorized within 13 of the 14 TDF domains. No codes were assigned to the domain ‘Belief About Capability’ (Table 2).

**Table 2:** Barriers ( −) and facilitators ( +) per TDF domain

*Capability*			
**Behavioural regulation and memory, attention and decision processes**	PN	GP	CP
Reminders of the combiconsultation during work	**+ **	**+ **	
Daily routine tasks take precedence	**− **	**− **	**− **
**Knowledge and skills**	PN	GP	CP
Sufficient pharmacotherapeutic knowledge of the pharmacist	**+ **	**+ **	**+ **
Insufficient consultation skills of the pharmacist		**− **	**− **
Insufficient clinical reasoning skills of the pharmacist		**− **	
*Opportunity*			
**Environmental context and resources**	PN	GP	CP
The pharmacist’s consultation room is in the general practice	**+ **	**+ **	**+ **
The pharmacist has access to medical data	**+ **	**+ **	**+ **
The healthcare providers have access to each other’s appointment ledger	**+ **		**+ **
Dependence on each other’s appointment ledger for scheduling consecutive consultations	**− **	**− **	**− **
Understaffed for scheduling consultations		**− **	**− **
Lack of consultation room for the pharmacist in the general practice	**− **	**− **	
**Social influences**	PN	GP	CP
A good existing collaboration between healthcare providers	**+ **	**+ **	**+ **
Patients appreciate extra attention about their medication	**+ **	**+ **	
Lack of alignment between PN and pharmacist regarding expectations of the CombiConsultation	**− **		
*Motivation*			
**Social/professional role and identity**	PN	GP	CP
The pharmacist’s role is to answer questions about medication			**+ **
The CombiConsultation improves the visibility of the pharmacist			**+ **
The pharmacist is a partner of the GP, with their own expertise		**+ **	
**Optimism**	PN	GP	CP
Belief in the care-providing role of the pharmacist	**+ **	**+ **	**+ **
**Beliefs about consequences**	PN	GP	CP
An improved contact between the pharmacist and the GP/PN	** + **	** + **	** + **
The established relationship with the patient			**+ **
The time saved compared to CMR			**+ **
The interventions identified by the pharmacist improves the quality of care	**+ **	**+ **	**+ **
The time saved for the PN during the periodic check-up	**+ **		
Healthcare providers learn from each other	**+ **	**+ **	
The patients’ acceptance of medication advice from the pharmacist	**+ **		
The selected patients do not all benefit from a CombiConsultation	**− **		**− **
An extra healthcare provider (pharmacist) requires more coordination		**− **	
The GP sometimes doubts the added value of the intervention proposals		**− **	
**Reinforcement**	PN	GP	CP
The CombiConsultation provides satisfaction	**+ **		**+ **
The reimbursement of the consultations is insufficient		**− **	**− **
**Intentions and goals**	PN	GP	CP
Healthcare providers desire the CombiConsultation to become routine in the future	**+ **	**+ **	**+ **
Difficulties in the continuation of the CombiConsultation in current daily practice			**− **
**Emotion**	PN	GP	CP
The CombiConsultation raises the PN to a higher level		**+ **	
It is satisfying to get the patient on correct medication	**+ **		
The pharmacist derives job satisfaction from contributing to the well-being of the patient			+

Bold: TDF domain

*PN*: practice nurse; *GP*: general practitioner; *CP*: community pharmacist; *CMR*: clinical medication review; + : facilitator; − : barrier

### Capability

In the Capability domain of COM-B, barriers and facilitators were found within the 4 underlying TDF domains below:

#### Behavioural regulation and memory, attention and decision processes

The analysis showed that all healthcare providers indicated that daily clinical practice always has priority. This opinion suggests that in their perception the CombiConsultation is not yet common practice.

“When people have questions about medication, you think “that’s great for the CombiConsultation”. (...) It was not unwillingness, but it [the CombiConsultation] was not on top of mind during the consultation.” *(PN 1)*

The PNs and GPs indicated that reminders of the CombiConsultation, such as a prompt via the GP system (‘patient is eligible for a consultation with the pharmacist’), would help to invite patients for a CombiConsultation:

“A pop-up from the GP system: this is a patient eligible for a polypharmacy consultation (…) helps to bring it to the attention of the doctor continuously.” *(GP3)*

#### Knowledge and skills

All healthcare providers considered CPs to have sufficient pharmaceutical knowledge. However, the GPs and CPs indicated that the pharmacists needed more consultation skills, and the GPs expressed some doubts regarding the clinical reasoning competence of the pharmacists:

“[The] pharmacist looks at certain complaints from a pharmacological perspective, while the GP may take a more generalist approach. (...) [The] pharmacist has a different background and certain knowledge that the PN lacks and the GP may not have immediately available either.” *(GP 4)*

### Opportunity

In the Opportunity domain of COM-B, barriers and facilitators were found within the two underlying TDF domains:

#### Environmental context and resources

With regard to ‘Environmental Context and Resources’, the main barriers and facilitators were related to access to information and working places to efficiently plan and perform CombiConsultations. According to all healthcare providers, access to medical data is a facilitator for performing CombiConsultations. Medical data helps to propose interventions that match patients’ needs. GPs also indicated that shielding certain conditions would be desirable:

“I think that what you need [to provide care] you should have access to.” *(GP 3)*

CPs and PNs found that access to each other’s appointment ledger could facilitate scheduling consultations.

“ICT can also contribute to this if you have a joint appointment ledger in which you can schedule [the consultations] and that is also simple and clear; that could make a difference.” *(CP 9)*

The interviewed CPs, GPs and PNs thought that the planning of consecutive consultations was a challenge, mainly due to different working hours, part-time work and insufficient staff:

“I couldn’t manage to schedule that [consultation with the pharmacist] consecutively. That was purely related to both providers’ working part-time.” *(PN 1)*

Some pharmacists indicated that access to a consultation room in the general practice ensures easy communication between healthcare providers and is a trusted environment for patients:

“I think it would be better if the pharmacist works in the GP setting. There are more contact moments [between healthcare providers].” *(CP 5)*

However, the PNs and GPs indicated that it takes considerable effort to find a suitable consultation room for the pharmacist in the GP’s practice due to lack of space. In addition, some pharmacists indicated that conducting the consultations in the pharmacy is also a good option, especially if the PN or GP works in the same building.

#### Social influences

The interviews showed that according to CPs, GPs and PNs, an existing collaborative practice facilitates the implementation of the CombiConsultation.

“In my opinion, having a confidential working relationship contributes to the confidence that things will work out [implementing the CombiConsultation]. That [trust] is fundamental.” *(GP4)*

A single PN indicated that lack of alignment between PN and CP regarding expectations of the CombiConsultation can be a barrier to performing CombiConsultations. The PNs and GPs indicated that patients were very satisfied with the extra attention for their medication:

“The patients to whom I introduced the CombiConsultation were very enthusiastic. Glad that someone takes a critical look at their medication and that there is special attention for it. It was really appreciated.“ *(PN 1).*

### Motivation

In the Motivation domain of COM-B, barriers and facilitators were found within 7 TDF domains, which are described below.

#### Social/professional role and identity

The interviewed pharmacists stated that answering questions regarding medication as part of their professional role and performing the CombiConsultations strengthened their roles as providers of pharmaceutical care. The data suggest that the GP certainly views the CP as a partner, with their own expertise, whom they can approach mainly for (practical) questions regarding medication. Although the participating GPs appreciated the pharmacists’ contributions to the pharmacotherapy, they expected that not every GP would be open to cooperation with a pharmacist:

“I notice that my colleagues sometimes think, “Stick to what you know.” The old idea of the traditional pharmacist, that he should not interfere with our work. (…) While I see us very clearly as partners in a safe medication world. He provides his part of the whole and we do our part.” *(GP 1)*

#### Optimism

The GPs, PNs and CPs expressed confidence in the care-providing role of the pharmacist and expected that the content of the profession would continue to develop in the future:

“I suspect that in the future the pharmacist will indeed be a pharmacotherapeutic consultant in the general practice rather than in the pharmacy itself. I would consider that as a good development.” *(GP 3)*

#### Beliefs about consequences

The analysis showed that CPs, GPs and PNs believed that the CombiConsultation had strengthened interprofessional collaboration and interprofessional learning. As a result, all interviewed healthcare providers believed that the pharmacotherapeutic interventions proposed by the CP during the CombiConsultation had improved the quality of pharmacotherapy.

“It is clear to me that it [the CombiConsultation] improves the quality [of care] (...) one patient is still very clear in my mind (...) He feels much better and is less at risk. He uses a lot less medication.” *(GP1)*

However, GPs sometimes questioned the clinical relevance of proposals and realized that an additional healthcare provider also required more coordination. In addition, the CPs and PNs thought that not all selected patients had benefitted from a CombiConsultation. The CPs experienced that the CombiConsultation had helped to build a stronger treatment relationship with the patient by allowing time to discuss their concerns and complaints regarding the medication. These opinions were in line with those of the PNs, who believed that patients had attached great value to the pharmacist’s medication advice:

“By having the conversation, you can build a relationship (...) you develop a relationship that gives them confidence. Not necessarily in you, but also in the drugs they take. And if there’s something they don’t trust, they’ll come to us [the pharmacists].” *(CP 5)*

The CPs experienced that performing the CombiConsultation took less time than a clinical medication review. The PNs also experienced time savings through the CombiConsultation (they spent less time on questions about medication), although planning of consultations could take more time:

“Sometimes they have so many questions, then you have to devote an extra consultation to the rest of the questions (...) So yes, it certainly fills a need.” *(PN 5)*

#### Reinforcement

Pharmacists and PNs reported receiving ‘interprofessional’ energy from conducting the CombiConsultation together.

“And everyone [all healthcare providers] is satisfied afterwards [of working together on a CombiConsultation], it was useful again.” *(PN4)*

However, GPs and CPs saw insufficient reimbursement as a large barrier for implementing the CombiConsultation:

“It’s very strange that when you do this job, you don’t get paid for it. (…) you can’t do it for free, I think. I would like it if there would be reimbursement from the health insurer.” *(CP 6)*

#### Intentions and goals

The GPs and PNs indicated that they wished the CombiConsultation to become routine in 5 years, and the CPs were prepared to give high priority to the implementation of CombiConsultation:

“I hope that in 5 years all our patients in chronic-disease-management programmes will have an annual CombiConsultation.” *(GP 1)*

#### Emotion

The data showed that the healthcare providers were enthusiastic about the CombiConsultation. The GPs appreciated that the CombiConsultation had lifted the PNs to a higher level. The PNs stated that it was satisfying to get the patient on the correct medication, and CPs were satisfied that they could contribute to the well-being of the patients. These emotions contributed to the motivation to conduct CombiConsultations:

“Especially what it [the CombiConsultation] has done to my PN. The fact that it has really lifted her to a much higher level, in terms of the enormous learning curve she went through there, I think that is the best outcome (…).” *(GP 1)*

## Discussion

Although the CombiConsultation is a promising intervention to improve safety and effectiveness of pharmacotherapy, implementation has proven difficult. The present study has identified 12 barriers and 23 facilitators that may influence the preparedness and willingness of healthcare providers to implement the CombiConsultation.

The CombiConsultation with the CP is integrated into the patient’s chronic disease management programme, which increases the involvement of the pharmacist in the treatment of the patient’s chronic condition. Our analysis found that all healthcare providers agreed that the CP is the appropriate professional to provide the CombiConsultation based on their expertise in medication. However, many also stated that the CP lacks sufficient consultation- and clinical-reasoning skills to perform the CombiConsultation optimally. This is consistent with conclusions of Hazen et al. They showed that pharmacists who work completely ‘embedded’ in a general practice experience difficulties with the transition from community-based, medication-focussed care to taking responsibility for the patient’s pharmacotherapy [19]. To prepare the pharmacist for this position, training in patient-centred care and clinical decision-making are therefore essential [20]. The non-dispensing pharmacists in the study of Hazen et al. were extensively trained [19]. However, The CombiConsultation study focussed on CPs, for whom extensive training was not feasible. It is important to investigate how pharmacists can be trained in this area. An example is adapting academic education by developing teaching strategies, like deliberate practice and feedback [21–23].

Our study also found that for all healthcare providers, their daily routine had retained priority over performing CombiConsultations. With regard to CPs, a previous study has shown that a substantial proportion of their time is dedicated to tasks that either are obligatory (checking prescriptions) or need to be performed due to lack of sufficient staffing (e.g. the dispensing process) [24]. Understaffing is currently a persistent problem in the entire healthcare sector [25, 26]. [27]In order to normalize the CombiConsultation (and consultations in general), the CP might therefore consider reorganizing processes in the community pharmacy, such as separating logistics from the CP’s role of providing patient care [24]. An example is the ‘Dutch hub and spoke’ model in which a central dispensing pharmacy (hub) supplies labelled medicines directly to satellite pharmacies (spokes) to allow the pharmacist to focus on pharmaceutical care [28]. GPs and PNs indicated that they needed to be reminded of the CombiConsultation, otherwise they would not think of referring patients to a pharmacist. Therefore, delegating tasks such as selecting and inviting patients is also essential and ensures more scheduled consultations.

Important preconditions for implementation of the CombiConsultation are access to medical data (at least conditions and laboratory values) [29] and access to each other’s appointment ledger. The latter is especially important for planning the consultations and communicating with the other healthcare providers (e.g. posting notes). In the current age of rapidly evolving information technology, ensuring the security, privacy and protection of patients’ healthcare data is critical [29, 30]. CPs and GPs should investigate the possibilities for shared access and possibly shielding of irrelevant (confidential) information from the pharmacist. As the Health Insurance Portability and Accountability Act and General Data Protection Regulation become stricter, this might cause more fear among healthcare providers related to ‘breaking the rules’ [30, 31]. However, limited access to patient medical data restricts the pharmacists’ ability to optimally contribute to the quality of pharmaceutical care [32]. Online access to medical data from the pharmacy might be more suitable; although it is challenging, it can often be arranged [33].

A consultation room in the general practice for the pharmacist can be a facilitator, as the pharmacists can work directly from the GP system (provided that clear agreements are made regarding patient confidentially), and it might be a safer environment for the patient to discuss their medication in the clinic. However, performing consultations at the GP’s site was also seen as a barrier because of limited space. Therefore, some CPs had conducted the CombiConsultations in the consultation room of the pharmacy; CPs whose pharmacies were located in the same building as the GP practices especially saw no obstacle in this regard. Co-location appeared to facilitate a greater level of integration into the primary health care team, and the benefits of co-location could also be achieved through regular face-to-face contact between health care professionals [34]. A workplace in practice is therefore not a strict requirement for being able to perform CombiConsultations. However, effective coordination related to the CP’s workplace and consultation availability with other healthcare providers is certainly crucial. In addition, professional respect and understanding of each other's role in providing patient care is an important factor in facilitating collaboration [35, 36]. By implementing CombiConsultations, CPs can fulfil a new role within primary care, providing a new professional identity. A general practice that values and accepts the new roles for the CP would likely enhance the process of role incorporation [37].

In addition, a healthcare institution in which all healthcare professionals work together enhances the professional image presented to patients and could make an additional contribution to build a relationship with the patient [38].

With respect to motivation, reimbursement is an important factor for both CPs and GPs. CPs are still predominantly reimbursed for dispensing, and in most countries there is no consistent way for pharmacists to obtain reimbursement for clinical pharmacy services [26]. However, reimbursement is essential for the widespread implementation of a clinical pharmacy service. Reimbursement for the provision of care will gradually increase, but in most countries this development is a slow process [39, 40].

### Strengths and limitations

A major strength of this study is the use of a theoretical model to underpin our data analysis. Another strength is that all categories of healthcare providers involved in the CombiConsultation were interviewed, resulting in a wide range of perspectives with high agreement between the healthcare providers. Since all invited healthcare providers agreed to participate, the use of incentives (voucher) to motivate the participants did not led to selection bias.

Although focus groups might have given more interaction between the participants, interviews were opted to achieve more depth and to collect experiences in a specific setting.

It should be noted that only healthcare providers participating in the CombiConsultation intervention study were interviewed. They are generally highly motivated and therefore not representative for all healthcare providers. However, in order to give a good representation of the experienced barriers and facilitators during the implementation of the CombiConsultation, experience with the CombiConsultation was essential. Also, we conducted the interviews both face-to-face as by telephone. Despite the fact that face-to-face interviews can theoretically provide more depth, this was not always feasible in terms of distance and time. In these cases, a telephone interview was conducted. However, we took this into account during analysis and we have no indications that there was a relevant difference between the two methods in our study.

A limitation of this study is that the interviewer and investigators were pharmacist or pharmacy student. This condition might have made other healthcare providers reluctant to share negative experiences with pharmacists. However, they still shared these experiences with the researchers.

## Conclusion

The current study has shed light on the high agreement of perspectives of healthcare providers regarding the implementation of the CombiConsultation. An existing collaborative practice, with a clear and accepted professional role of the pharmacist is essential for implementation. Training of pharmacists in consultation-and clinical-reasoning skills can be beneficial, as well as arrangements on the consultation logistics, sufficient staff and reimbursement.

## Supplementary Information

Below is the link to the electronic supplementary material.

Supplementary file1 (DOCX 28 kb)
