# Supplementary material for: Barriers and facilitators for the implementation of the CombiConsultation by general practitioners, pharmacists and practice nurses: a qualitative interview study
Source: Int J Clin Pharm. 2023 May 30;45(4):970–9. doi: 10.1007/s11096-023-01597-7 (PMC10366006; doi:10.1007/s11096-023-01597-7)
Supplement: Supplementary file 1 — Supplementary file1 (DOCX 28 kb) [file 11096_2023_1597_MOESM1_ESM.docx]

# Supplementary information 1: Interview Guides

## Pharmacist

| **Introduction**  Introduction, interview goal, approach, anonymity, voice recorder, duration |
| --- |
| **General questions**   - How did the last CombiConsultations go?   - Positive/negative   - If it's been a long time: Do you miss it? Why/why not? |
| **Knowledge/Skills**  *Did you have sufficient expertise to carry out CombiConsultations?*  *Were you sufficiently prepared/trained to carry out CombiConsultations?*   - Procedure/organization - Sufficient training? - Pharmacotherapeutic knowledge/clinical reasoning - Communication/consultation - Collaboration and organization |
| **Role/Identity**   - Do you think performing CombiConsultations is an appropriate task for the community pharmacist? Why/why not?   - Is performing CombiConsultations part of your expertise?   - Can someone else also do this? - How did the CombiConsultation affect your relationship with other healthcare providers? - What is your role in relation to the PN? And the GP? |
| **Beliefs About Consequences/Reinforcement**   - When did you think you had helped a patient? - For which group of patients have you been able to mean the most? - What does the CombiConsultation offer you? |
| **Environmental Context and Resources**   - What do you need from the environment [stakeholders: health insurance, organization, etc.] to implement the CombiConsultation?   - Funding was available. Why then did we not manage to reach the inclusion number?   - Organization/time   - Other care providers   - What do you think is the added value of conducting the consultations in the GP practice or pharmacy? |
| **Social Influences**   - Were you supported by others in implementing/performing the CombiConsultation?   - Fellow pharmacists   - Pharmacy technicians   - PN   - GP   - Patients |
| **Emotions**   - What were your high points in this project (executing CombiConsultations)? - What were your low points in this project (executing CombiConsultations)? - How did you feel when you had to schedule consultations? - How did you feel when you knew that a CombiConsultation was scheduled for that day? |
| **Intentions/Goals**   - What is the main reason for you to perform CombiConsultations? - If you had to rank all your activities by priority, where would you rank the CombiConsultation? - Are there any intentions to continue the CombiConsultation?   - If so, in what form?     - Time to invest? Frequency? Which patient group? With which healthcare providers?   - If not, why not? - What would you like to achieve in 5 years with the CombiConsultation? |
| **Beliefs About Capabilities/Optimism**   - Are you confident that you can put the CombiConsultation into practice on a structural basis?   - What contributes to that confidence?   - Who or what hinders that trust?   Patients/care providers/organization |

## Practice nurse

| **Introduction**  Introduction, interview goal, approach, anonymity, voice recorder, duration |
| --- |
| **General questions**  What is your general impression of the CombiConsultation? |
| **Knowledge/Skills**   - Do you pay attention to medication during your consultation? To what extent can you participate in the advice that the pharmacist provided about the medication?      - How did you structure the consultation with the pharmacist before/after the consultation? |
| **Role/Identity**   - What were your first thoughts about your role (practice nurse) when you heard about the CombiConsultation?   - To what extent did those thoughts come true? - What do you think is the value of the pharmacist in this collaboration? |
| **Beliefs About Consequences/Reinforcement**   - Does the quality of the pharmacotherapy improve as a result of the combination consultation? Can you provide an example? - In what way does the CombiConsultation contribute to the treatment of the patient or to his/her quality of life? - To what extent has the collaboration with the pharmacist changed during the CombiConsultation? - Has your consultation been changed by the CombiConsultation? Do you conduct your consultations differently now? Can you provide an example? |
| **Environmental Context and Resources**   - What do you need from your environment (Time, organization, ICT)? |
| **Social Influences**   - Do you feel supported by others when implementing/executing the CombiConsultation in practice?   - Fellow PNs: Were colleagues enthusiastic about starting the CombiConsultation?   - Pharmacist   - Pharmacy technicians   - Patients - How does the CombiConsultation affect your relationship with other healthcare providers, especially the pharmacist? - What was the influence of the previous collaboration with the pharmacist on the implementation of the CombiConsultation? |
| **Emotions**   - What were your high points in this project (executing CombiConsultations)? - What were your low points in this project (executing CombiConsultations)? - How did you feel when you had to schedule consultations? - How did you feel when you knew that a CombiConsultation was scheduled for that day? |
| **Intentions/Goals**   - What is the main reason for you to perform CombiConsultations/that the practice is involved in the CombiConsultation? (Collaboration/helping the patient) - Are there any intentions to continue with the CombiConsultation?   - If so, in what form?     - How much time to invest? Frequency? Which patient group? Which healthcare providers?   - If not, why not? - What would you like to achieve in 5 years with the CombiConsultation? |
| **Beliefs About Capabilities/Optimism**   - Are you confident that you can put the CombiConsultation into practice on a structural basis?   - What contributes to that confidence?   - Who or what hinders that trust?   Patients/care providers/organization |

## General practitioner

| **Introduction**  Introduction, interview goal, approach, anonymity, voice recorder, duration |
| --- |
| **General question**  What is your general impression of the CombiConsultation? |
| **Knowledge/Skills**   - Did you feel that pharmacist, general practitioner and practice nurse had the knowledge and skills needed to successfully implement the CombiConsultation?   - What knowledge do you lack? Can the pharmacist contribute to this? |
| **Role/Identity**   - Has the CombiConsultation changed your view of ‘the pharmacist as a healthcare provider’? If so, how? - What do you think is the role of the pharmacist in the CombiConsultation?   - Focussed on pharmacotherapy/adherence/medication use?   - Will the role of the GP change due to the CombiConsultation (pharmacist as healthcare provider)? If so, how?   - What other possibilities do you see for closer collaboration between pharmacist and GP? |
| **Beliefs About Consequences/Reinforcement**   - In what way does the CombiConsultation contribute to the treatment of the patient or to his/her quality of life? - Was the investment in collaboration with the pharmacist worthwhile during the CombiConsultation? Can you provide an example? - How should pharmacists be valued for these activities? |
| **Environmental Context and Resources**   - What do you need from your environment (stakeholders: health insurance, organization,etc.) to continue the CombiConsultation in practice?   (Finance, organization/time, other caregivers, etc.)   - What’s your view of the pharmacist who has access to medical data?   - What data should the pharmacist be able to view? |
| **Social Influences**   - Do you feel supported by others when implementing/executing the CombiConsultation in practice?   - Fellow GPs: Were colleagues enthusiastic about starting the CombiConsultation?   - PNs/technicians/patients - How does the CombiConsultation affect your relationship with other healthcare providers, especially the pharmacist? - What was the influence of the previous collaboration with the pharmacist on the implementation of the CombiConsultation? |
| **Emotions**   - What were your high points in this project (executing CombiConsultations)? - What were your low points in this project (executing CombiConsultations)? - How did you feel when you knew a CombiConsultation was scheduled for that day? |
| **Beliefs About Capabilities/Optimism**   - Are you confident that you can put the CombiConsultation into practice on a structural basis?   - What contributes to that confidence?   - Who or what hinders that trust?   Patients/care providers/organization/etc. |
| **Intentions/Goals**   - What is the main reason for you to perform CombiConsultations/that the practice is involved in the CombiConsultation? (Collaboration/helping the patient/etc.) - How has your acceptance of the CombiConsultation changed during the project? How did that happen? - Are there any intentions to continue with the CombiConsultation?   - If so, in what form?     - How much time to invest? Frequency? Which patient group? Which healthcare providers?   - If not, why not? - What would you like to achieve in 5 years with the CombiConsultation? |
